# Mind the “CRP gender gap”! sex differences in CRP evolution over time in neonatal sepsis: a monocentric retrospective cohort study

**DOI:** 10.1186/s13293-026-00841-9

**Published:** 2026-02-13

**Authors:** Andrea Nebbioso, Isaline Eggermont, Alfredo Vicinanza, Phu-Quoc Lê, Nancy Vitali, Gwenaëlle Augé, Nicolas Lefèvre

**Affiliations:** 1 Department of Paediatrics, Hôpital Etterbeek-Ixelles IRIS-sud, Rue Jean Paquot 63, 1050 Bruxelles, Belgium; 2 Department of Paediatrics, Université libre de Bruxelles (ULB), Hôpital Universitaire de Bruxelles (H.U.B), Hôpital Universitaire des Enfants Reine Fabiola (HUDERF), Avenue J.J. Crocq 15, 1020 Bruxelles, Belgium

**Keywords:** CRP, Neonatal sepsis, Neonatal infection, Neonatal inflammation, Inflammation, Sex differences, Sexual dimorphism

## Abstract

**Background:**

C-reactive protein (CRP) is a readily available test widely used to assess neonatal sepsis (NS). In children with sepsis or other infectious conditions, CRP is more likely to be higher in females than males, however, evidence is lacking on sex differences in CRP in the neonatal population. This study aims to describe sex differences of CRP evolution in the ascending and decreasing phase after its peak in neonates with likely NS.

**Methods:**

This is a monocentric retrospective cohort study conducted at Etterbeek-Ixelles Hospital in Brussels. We included all neonates born in the facility between January 2017 and December 2022 who received antibiotics in the first 72 hours of life. Patients whose CRP concentrations remained under 10 mg/L were excluded. To describe the ascending kinetics of CRP and its logarithm for male and female neonates, we fitted a piecewise linear mixed-effects regression model with birth considered as time zero and one knot at 12 hours of life. We used a linear mixed-effects regression model with CRP peak considered as time zero to describe CRP’s descending kinetics and its logarithm for male and female neonates.

**Results:**

We included 506 neonates (60.1% male and 39.9% female). CRP concentration in the first 12 hours of life doubled every 3.2 and 2.8 hours, respectively, in males and females, with female neonates having a statistically significant faster rise of base 2 logarithm of CRP (+0.04 log2 mg/L/hour 95% CI= +0.01 +0.07). After 12 hours of life, CRP doubled every 6.5 and 8.6 hours, respectively, in males and females, with female neonates having a statistically significant slower rise of base 2 logarithm of CRP (-0.039 log2 mg/L/hour 95% CI= -0.02 -0.06). After its peak, CRP decreased by half every 31.1 and 30.9 hours, respectively, for males and females. No statistically significant sex differences were found in CRP peak or decline.

**Conclusion:**

In neonates of both sexes with likely but unconfirmed NS, CRP seems to increase, reach a peak, and then decrease, following a logarithmic pattern. Before antibiotic treatment, female neonates in our population showed an earlier increase in CRP levels, with no difference in peak CRP levels.

**Supplementary Information:**

The online version contains supplementary material available at 10.1186/s13293-026-00841-9.

## Plain language summary

C-reactive protein (CRP) is produced by the liver during inflammation and is widely measured to assess a range of infectious and inflammatory conditions. In children, CRP levels are often higher in females than in males, but data on sex differences in the neonatal period (0–28 days) are limited. This study aims to characterize sex differences in CRP concentrations among neonates with suspected infection. We examined both phases of CRP kinetics: the rising phase, reflecting the host response before treatment, and the declining phase, reflecting recovery following antibiotic therapy. A total of 506 neonates were included in the study (304 male neonates and 202 female neonates). Before treatment, female neonates showed an earlier increase in CRP, although peak levels did not differ significantly between sexes. In the first 12 hours of life, CRP doubled every 3.2 hours in males and every 2.8 hours in females, indicating a faster rise in females. After 12 hours, the doubling time was 6.5 hours in males and 8.6 hours in females, suggesting a slower rate of increase in females. No sex differences were observed in the declining phase of CRP. Our findings highlight that immune responses differ by sex even in the neonatal period. Clinicians should be aware that sex is a relevant factor when interpreting standard laboratory markers such as CRP.

**Supplementary Information:**

The online version contains supplementary material available at 10.1186/s13293-026-00841-9.

## Highlights

Compared to male neonates, female neonates of our population showed a statistically significant earlier rise in C-reactive protein (CRP) levels before antibiotic treatment.

CRP probably increases following a logarithmic pattern with concentrations of the first 12 hours of life doubling every 3.2 hours for male neonates and every 2.8 hours for female neonates. Therefore, the speed of exponential growth decreases after the 12^th^ hour of life with a doubling time of 6.5 hours for male neonates and 8.6 hours for female neonates.

Despite earlier rise in CRP level for female neonates, we found no significant difference between sexes in CRP peak levels.

After reaching its peak, CRP decreases following a logarithmic pattern, dropping by half every 31.1 hours in males and 30.9 hours in females, with no statistically significant difference between sexes.

**Supplementary Information:**

The online version contains supplementary material available at 10.1186/s13293-026-00841-9.

## Introduction

Neonatal sepsis (NS) has been defined as a systemic condition of infectious origin, resulting in substantial morbidity and mortality in neonates [1]. Most authors consider the first 72 hours of life the cut-off between early and late-onset NS, while others refer to late-onset NS (LONS) as occurring after seven days of life [1]. C-reactive protein (CRP) is the most widely used and readily available laboratory test to assess NS [2]. Two CRP values <10 mg/L obtained 24 hours apart have very high specificity and, therefore, strong power in excluding early-onset NS (EONS) [3]; however, once the clinician faces the opposite situation (one CRP measurement >10 mg/L), CRP cut-offs multiply and sensitivity drops below 70% [4]. Previous studies demonstrated that CRP values in healthy neonates are log-normally distributed and physiologically rise in the first 24 hours of life starting from a very low level at birth [5] as CRP does not pass through placenta [6]. No sex differences in neonatal CRP levels have been found in a healthy neonatal population [5]. Many studies focused on the specificity and sensibility of CRP at specific times for the diagnosis of NS [4]. The evolution of CRP over time in neonates with NS or other inflammatory conditions has been poorly described. One study, with different primary goals, accidentally described CRP values over time in neonates with NS with few cases of confirmed NS, mostly in preterm, with only 4 cases of term neonates [7]. Another study found higher CRP velocity in females, but they included also neonates with normal CRP levels, neonates not receiving antibiotics and they did not specify the sampling time and whether CRP measurement were done before or after antibiotic start [8]. Until now, no study has been published describing CRP values over time after antibiotic treatment in the neonatal population. The CRP decrease pattern over time after its initial peak has been described mainly in the adult population with an estimated half-life of 19 hours [9]. We found no neonatal-specific data assessing CRP half-life after its peak. Increasing evidences suggest that overlooking sex differences in research leads to poorer outcomes for females [10, 11]. A large epidemiological study on severe sepsis in children in the United States confirmed that being male is a risk factor for worse outcomes in cases of prematurity and some neonatal infections [12]. Moreover, a genome-wide association study with 224 LONS cases showed that genetic susceptibility to LONS exhibits sexual dimorphism, with NOTCH signaling playing a role in determining the associated risk [13]. In children with sepsis or other infectious conditions, CRP values are more likely to be higher in females than in males [14, 15]. In the early stage of the infection, inflammatory biomarkers are more elevated in females, indicating a stronger immune activation. Over time, a distinct kinetic pattern emerges between sexes in the return to homeostasis. In contrast, levels of proinflammatory cytokines, such as interleukin (IL)−1β, and tumor necrosis factor-alpha (TNF-α), are higher in men, whereas women exhibit elevated levels of anti-inflammatory mediators, notably IL-10 [16–19].

This study aims to determine whether there are sex-based differences in CRP kinetics over time during the early neonatal period in neonates with likely EONS. Our objective is to provide sex-based differential references describing how CRP rises before and stabilizes after antibiotic treatment and how quickly CRP decreases after its peak.

## Methods

### Operational definitions

Early onset neonatal sepsis (EONS): NS occurring within the first 72 hours of life [1].

Clinical symptoms: Fever (> 38°), hypothermia (< 36,0°), poor sucking, hemodynamic instability, apnoea, respiratory distress, cyanosis, grunting, irritability, recurrent hypoglycemia, and purpura-like cutaneous rash.

Infectious risk factors: maternal fever, prolonged membrane rupture, maternal group B streptococcus, colonization, chorioamnionitis and maternal antibiotic treatment + integrated calculation of risk using neonatal sepsis risk calculator, as developed by Kuzniewicz et al. [20–23].

Abnormal laboratory findings: CRP higher than 20 mg/L or white cells lower than 4000/μl.

Suspected EONS: neonates treated with antibiotics because the clinician suspected EONS on the basis a combination of at least two of the following three criteria: presence of at least one clinical symptom, one infectious risk factor and one abnormal laboratory finding.

Unlikely EONS: suspected EONS (as previously defined) with CRP measurements never higher than 10 mg/L.

Likely EONS: suspected EONS (as previously defined) with at least one CRP measurement higher than 10 mg/L.

Confirmed EONS: suspected EONS with positive culture of blood or another sterile site.

Unconfirmed EONS: culture-negative suspected EONS.

### Study design and setting

This is a monocentric retrospective cohort study conducted in the neonatal unit (NU) and maternity ward (MW) of Etterbeek-Ixelles Hospital (EIH). We included all neonates born in the facility between January 2017 and December 2022 and treated with antibiotics. We excluded patients who started antibiotic therapy after 72 hours of life. Patients whose CRP concentrations remained under 10 mg/L during the hospital stay (unlikely EONS) were also excluded. Only blood samples obtained in EIH were considered in the analysis of patients who had to be transferred to other centres. EIH MW has an average of 2000 live births/year, and the NU has an admission rate of approximately 400 patients/year with a multi-ethnic patient population. Neonates treated with antibiotics in EIH MW are systematically admitted for the first few hours in the NU. As EIH NU provides sub-intensive neonatal care, preterm neonates before 32 weeks of gestational age are transferred to larger centres, as well as neonates with a birth weight under 1500 grams. EIH NU has incubators and can provide non-invasive ventilation, oxygen therapy, parenteral nutrition via central lines and nasogastric tube feeding. In EIH NU and MW, the first-line treatment of NS is a combination of amoxicillin-amikacin (5 days of amoxicillin and 2 days of amikacin), and the second-line treatment consists of the association of amoxicillin-cefotaxime. The tool of the neonatal sepsis risk calculator, as developed by Kuzniewicz et al*.* [20–23] has been systematically used to assess the NS risk since January 2020. In EIH NU and MW, CRP is measured before antibiotic treatment at 0, 12, and 24 hours of life in the presence of infectious risk factors. CRP is systematically measured 12 and 30 hours after antibiotic treatment. EIH laboratory uses an immunoturbidimetric method by Cobas 8000 module c702 of Roche Diagnostics in order to measure CRP. The detection limit is 0.3 mg/l, the inter-assay coefficient of variation is 4,93% at a concentration of 0,84 mg/L (standard deviation: 0,04 mg/L), based on local method verification using internal quality controls.

### Data collection and statistical analysis

Patients’ files were retrospectively reviewed to collect the data. The following information was collected: date of birth, birth weight and its Z-score, gestational age at birth, sex, type of delivery, presence of infectious risk factors, data about clinical presentation of neonates (fever, jaundice, respiratory distress, time of antibiotic start and blood culture result), CRP values and time of sampling. Frequencies and proportions were calculated for categorical variables. The Fisher’s exact test was used to determine the association between categorical variables. Continuous variables were described using the median and interquartile range. The Mann-Whitney test was performed for continuous variables. By convention, we decided to express time in hours and CRP in mg/L. We will refer to “base 2 CRP logarithm” (CRPlog2) as the base 2 logarithm of CRP values expressed in mg/L. We decided to describe CRP and CRPlog2 kinetics according to three different time axes. The first axis of time considers birth as time 0 and aims to describe the ascending phase of CRP and CRPlog2 before antibiotic start. The second axis of time considers antibiotic start as time 0 and seeks to describe the ascending-descending transition phase. The third axis of time designates time 0 as the point at which CRP peaks and aims to describe the subsequent descending phase. We fitted two identical models (one for CRP and one for CRPlog2) for each time axis. To describe the ascending kinetics of CRP and CRPlog2 in male and female neonates, we fitted a piecewise linear mixed-effects regression model where birth was considered as time zero with one knot at 12 hours of life, with observations occurring after 24 hours of life or after antibiotic treatment being excluded. Sex was the only covariate in this model. To describe the transition of CRP and CRPlog2 from ascending to descending phase, we fitted a piecewise linear mixed-effects regression model where antibiotic treatment start was considered as time zero with one knot at 12 hours of antibiotic treatment and observations occurring before antibiotic treatment or 30 hours after were excluded. Sex and time of antibiotic start (at birth vs after birth) were the two covariates in this model. To describe the descending kinetics of CRP and CRPlog2 in male and female neonates, we fitted a linear mixed-effects regression model where the CRP peak was considered as time zero, with observations occurring before the CRP peak or 120 hours after being excluded. Sex was the only covariate in this model. In all fitted models, we introduced a random effect on intercepts and slopes and we used the restricted maximum likelihood method to calculate standard error. We used an unstructured covariance matrix for serial correlation for all fitted models. A p-value of < 0.05 was considered statistically significant. Statistical analyses were performed using STATA version 19 for MacOS (StataCorp, College Station, Texas 77845 USA).

## Results

During the study period, 506 neonates met the inclusion criteria, with 304 males (60.1%) and 202 females (39.9%). Table 1 summarizes the population’s general demographic and clinical characteristics according to sex.

**Table 1:** General characteristics of neonates with CRP rise higher than 10 mg/L admitted to Ixelles neonatal unit (Bruxelles, Belgium) from January 2017 to December 2022

	Males (n=304)	Females (n=202)	Total (n=506)	Fisher’s exact/Mann-Whitney test p-value
**Neonatal characteristics**				
**Gestational age (n=506)**				
Late preterm (32–37 weeks)	10 (3.3%)	3 (1.5%)	13 (2.6%)	0.17
Term (>37 weeks)	294 (96.7%)	199 (98.5%)	493 (97.4%)	
Gestational age weeks (median p25 p75)	40 (39 41)	40 (39 41)	40 (39 41)	0.58
**Birth weight (n=506)**				
Low birth weight (1.5–2.5 kg)	11 (3.6%)	3 (1.5%)	14 (2.8%)	0.17
Normal birth weight (>2.5 kg)	293 (96.4%)	199 (98.5%)	492 (97.2%)	
Birth weight Kg (median p25 p75)	3.6 (3.23 3.86)	3.43 (3.16 3.73)	3.52 (3.2 3.8)	0.006
Birth weight Z-score (median p25 p75)	0.10 (−0.48 0.79)	0.16 (−0.5 0.81)	0.12 (−0.48 0.79)	0.34
**Clinical characteristics**				
Respiratory distress (n=499)	65 (21.7%)	44 (22.1%)	109 (21.8%)	0.91
Jaundice (n=499)	36 (12.0%)	13 (6.5%)	49 (9.8%)	0.047
Fever (n=503)	28 (9.3%)	19 (9.4%)	47 (9.3%)	1.0
Treatment started at birth (n=506)	86 (28.3%)	63 (31.2%)	149 (29.4%)	0.49
Treatment duration <72 hours (n=506)	12 (3.9%)	8 (3.9%)	20 (3.9%)	1.0
Positive blood culture (n=506)	0 (0%)	0 (0%)	0 (0%)	1.0
Contaminant positive blood culture (n=506)	7 (2.3%)	4 (2.0%)	11 (2.2%)	
**Delivery characteristics**				
**Delivery type (n=506)**				
Vaginal	141 (34.9%)	99 (49.0%)	240 (47.4%)	0.76
Vacuum-assisted	106 (46.4%)	70 (34.7%)	176 (34.8%)	
Caesarean	57 (18.7%)	33 (16.3%)	90 (17.8%)	
**Infectious risk factors**				
Maternal fever (n=491)	56 (19.0%)	44 (22.3%)	100 (20.4%)	0.42
Maternal chorioamnionitis (n=500)	8 (2.7%)	2 (1.0%)	10 (2.0%)	0.33
Prolonged membrane rupture (>18 h) (n=490)	68 (23.0%)	38 (19.5%)	106 (21.6%)	0.37
Meconial amniotic liquid (n=476)	113 (39.5%)	87 (45.8%)	200 (42.0%)	0.18
Maternal antibiotic treatment (n=499)	154 (51.5%)	86 (43.0%)	240 (48.1%)	0.07
GBS colonisation (n=482)	84 (29.2%)	42 (21.6%)	126 (26.1%)	0.07

In our population, 2.6% of neonates were born preterm, and 2.8% presented with a low birth weight. Treatment was started at birth in 29.4% of cases. Male neonates presented more frequently jaundice (12% vs 6.5%, p-value 0.047). We had no neonate with positive blood culture or fatal outcome. Table 2 shows the evolution of CRP and its base 2 logarithm before antibiotic treatment, in the first 12 hours of life and from 12 to 24 hours of life.

**Table 2:** Piecewise mixed linear spline regression model with random effects on intercept and slopes predicting CRP and base 2 CRP logarithm in male and female neonates from birth to antibiotic start in the first 24 hours of life

Fixed effects		CRP (mg/L)				CRPlog2 (mg/L)			
		Coefficient	P-value	[95% Conf. Interval]		Coefficient	P-value	[95% Conf. Interval]	
♂	Intercept (at birth)	2.177	<0.001	1.411	2.943	−0.563	<0.001	−0.758	−0.367
♀		2.338	<0.001	1.411	3.266	−0.522	<0.001	−0.758	−0.286
Delta ♀-♂		0.161	0.793	−1.042	1.363	0.040	0.796	−0.266	0.347
♂	h0-12 slope (/h)	0.907	<0.001	0.778	1.036	0.316	<0.001	0.300	0.334
♀		1.245	<0.001	1.086	1.404	0.355	<0.001	0.333	0.377
Delta ♀-♂		0.338	0.001	0.133	0.542	0.039	0.007	0.011	0.068
♂	h12-24 slope (/h)	2.064	<0.001	1.916	2.213	0.155	<0.001	0.142	0.168
♀		1.908	<0.001	1.718	2.097	0.116	<0.001	0.100	0.132
Delta ♀-♂		−0.157	0.202	−0.397	0.084	−0.039	<0.001	−0.060	−0.018

Random effects									
Variance of intercept		29.29		.	.	2.599		.	.
Variance of slopes									
H0-12		0.639		.	.	0.015		.	.
H12-24		0.784		.	.	0.005		.	.
Conditional intraclass correlation		0.68		0.68	068	0.93		0.93	0.93
Conditional R^2^		0.96				0.97			
Marginal R^2^		0.59				0.76			
Number of patients		497				497			
Number of observations		1042				1042			

Residual-fitted values diagnostic plots for both models are provided in supplementary materials section

Slopes are expressed in mg/L/hour. Intercepts are expressed in mg/L

♂: male neonates; ♀: female neonates Delta ♀-♂: difference between male and female neonates

**Fig. 1:**
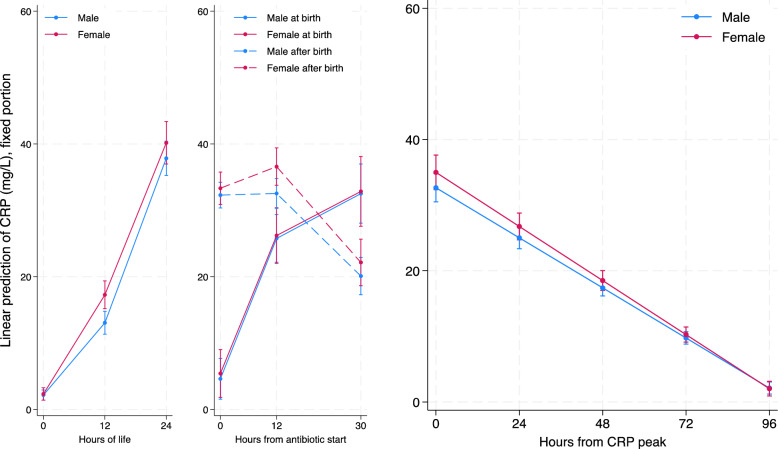
Graphic representation of CRP concentrations according to regressions models shown in tables 2 (left part), 3 (middle part) and 4 (right part). On the left part: Piecewise mixed linear spline regression model with random effects on intercept and slopes predicting CRP (mg/l) in male and female neonates from birth to antibiotic start in the first 24 hours of life (497 patients, 1042 observations). In the middle part: Piecewise mixed linear spline regression model with random effects on intercept and slopes predicting CRP (mg/l) from antibiotics start to 30 hours thereafter in male and female neonates according to the timing of antibiotics start (506 patients, 1525 observations). On the right part: Mixed linear regression model with random effects on intercept and slope predicting CRP (mg/l) in male and female neonates from CRP peak to 120 hours thereafter (506 patients, 1730 observations)

At time 0 (birth) both sexes had mean CRP values statistically different from 0 (2.18 mg/L; 95% CI= 1.41 2.94 for males and 2.34 mg/L; 95% CI 1.41 3.27 for females) with no statistically significant difference between sexes. In both sexes, CRP level rose in the first 12 hours of life (0.91 mg/L/hour; 95% CI= 0.78 1.04 for males and 1.24 mg/L/hour; 95% CI= 1.09 1.4 for females). Female neonates had a statistically significant faster rise in CRP (+ 0.34mg/L/hour; 95% CI= 0.13 0.54) compared to males. From hour 12 to hour 24 of life, CRP had a more rapid rise in both sexes (2.06 mg/L/hour; 95% CI=1.92 2.21 for males and 1.91 mg/L/hour; 95% CI= 1.72 2.1 for females) with no statistically significant difference between sexes. Figure 1 graphically represents these findings in its left part.

**Fig. 2:**
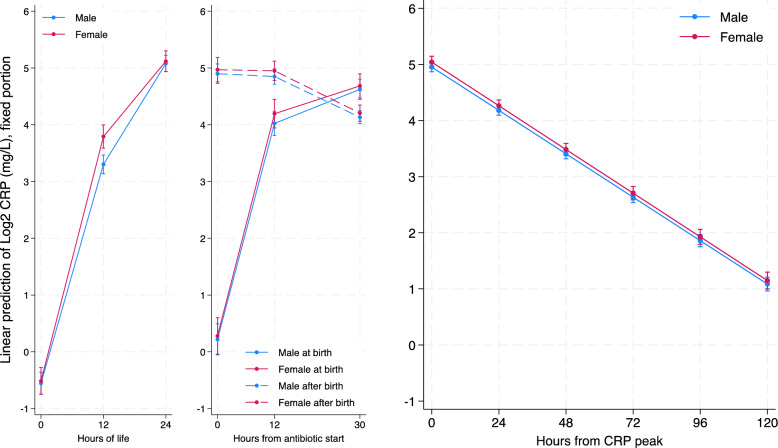
Graphic representation of CRPlog2 according to regressions models shown in tables 2 (left part), 3 (middle part) and 4 (right part). On the left part: Piecewise mixed linear spline regression model with random effects on intercept and slopes predicting base 2 CRP logarithm (mg/l) in male and female neonates from birth to antibiotic start in the first 24 hours of life (497 patients, 1042 observations). In the middle part: Piecewise mixed linear spline regression model with random effects on intercept and slopes predicting base 2 CRP logarithm (mg/l) from antibiotics start to 30 hours thereafter in male and female neonates according to the timing of antibiotics start (506 patients, 1525 observations).On the right part: Mixed linear regression model with random effects on intercept and slope predicting base 2 CRP logarithm (mg/l) in male and female neonates from CRP peak to 120 hours thereafter (506 patients, 1730 observations)

For CRPlog2, the pattern was quite similar, with no statistically significant difference between sexes at birth, and with both sexes having a mean CRPlog2 level different from 0 (−0.56 log2 mg/L; 95% CI=−0.76 −0.37 for males and −0.52 log2 mg/L; 95% CI= −0.76 −0.29 for females). In the first 12 hours, both sexes had a statistically significant rise in CRPlog2 (0.32 log2 mg/L/hour; 95% CI= 0.31 0.35 for males and 0.36 log2 mg/L/hour; 95% CI=0.33 0.38 for females) with a statistically significant faster rise of CRPlog2 in females (+0.039 log 2 mg/L/hour 95% CI= 0.01 0.07). From hour 12 to hour 24 both sexes had a statistically significant increase in CRPlog2 (0.15 log2 mg/L/hour; 95% CI= 0.14 0.17 for males and 0.12 log2 mg/L/hour; 95% CI=0.10 0.13 for females) with a less rapid rise of CRPlog2 in females (−0.039/hour 95% CI= −0.06 −0.018). This means that, in our population, CRP plasma concentrations in the first 12 hours of life doubled every 3.2 hours for male neonates and every 2.8 hours for female neonates. Therefore, after the 12^th^ hour of life, it took 6.5 hours for male neonates and 8.6 hours for female neonates to double CRP plasma concentrations. Figure 2 graphically represents these findings in its left part.

Table 3 shows the different evolutions of CRP levels and its base 2 logarithm after antibiotic treatment, according to sex and the timing of antibiotic treatment (started at birth vs not); slopes are described in the first 12 hours of antibiotic treatment and afterward, up to 30 hours from antibiotic start.

**Table 3:** Piecewise mixed linear spline regression model with random effects on intercept and slopes predicting CRP and base 2 CRP logarithm in male and female neonates according to the timing of antibiotics start (at birth or after) and from antibiotics start to hour 30 after its start

Fixed effects		CRP (mg/L)				CRPlog2 (mg/L)			
		Coefficient	P-value	[95% Conf. Interval]		Coefficient	P-value	[95% Conf. Interval]	
Antibiotics started at birth									
♂	Intercept (at antibiotic start)	4.61	0.003	1.55	7.68	0.219	0.116	−0.054	0.492
♀		5.42	0.003	1.82	9.03	0.279	0.088	−0.041	0.598
Delta ♀-♂		0.81	0.737	−3.92	5.54	−0.060	0.78	−0.360	0.480
♂	h0-12 slope (/h)	1.76	<0.001	1.47	2.06	0.317	<0.001	0.297	0.337
♀		1.73	<0.001	1.39	2.07	0.326	<0.001	0.303	0.350
Delta ♀-♂		−0.03	0.892	−0.48	0.42	0.0093	0.560	−0.022	0.041
♂	h12-30 slope (/h)	0.38	<0.001	0.17	0.58	0.033	<0.001	0.0257	0.041
♀		0.37	0.003	0.13	0.61	0.027	<0.001	0.0182	0.036
Delta ♀-♂		−0.006	0.971	−0.32	0.31	−0.0061	0.309	−0.0179	0.0057
Antibiotics started after birth									
♂	Intercept (at antibiotic start)	32.30	<0.001	30.36	34.24	4.900	<0.001	4.727	5.070
♀		33.33	<0.001	30.90	35.76	4.969	<0.001	4.754	5.184
Delta ♀-♂		1.04	0.514	−2.08	4.15	0.071	0.616	−0.205	0.346
♂	h0-12 slope (/h)	0.02	0.821	−0.16	0.20	−0.0041	0.531	−0.0168	0.0087
♀		0.27	0.02	0.04	0.50	−0.0015	0.856	−0.0175	0.0145
Delta ♀-♂		0.25	0.095	−0.04	0.54	0.0027	0.804	−0.0178	0.023
♂	h12-30 slope (/h)	−0.69	<0.001	−0.82	−0.56	−0.040	<0.001	−0.0447	−0.0351
♀		−0.80	<0.001	−0.96	−0.64	−0.041	<0.001	−0.047	−0.035
Delta ♀-♂		−0.11	0.293	−0.32	0.10	−0.00157	0.687	−0.0092	0.0061

Random effects									
Variance of intercept		83.95		62.34	113.07	1.57		1.369	1.802
Variance of slopes									
H0-12						0.008		0.006	0.009
H12-30						0.0005		0.0002	0.001
H post-antibiotic		0.09		0.04	0.17				
Conditional intraclass correlation		0.39		0.31	0.48	0.93		0.89	0.96
Conditional R^2^		0.67				0.96			
Marginal R^2^		0.20				0.62			
Number of patients		506				506			
Number of observations		1525				1525			

Residual-fitted values diagnostic plots for both models are provided in supplementary materials section

Slopes are expressed in mg/L/hour. Intercepts are expressed in mg/L

♂: male neonates; ♀: female neonates Delta ♀-♂: difference between male and female neonates

For neonates with antibiotics started at birth, at time 0 (antibiotic start and birth), both sexes had mean CRP values different from 0 (4.61 mg/L; 95% CI= 1.55 7.68 for males and 5.42 mg/L; 95% CI 1.82 9.03 for females). In both sexes, CRP levels rose in the first 12 hours of antibiotic treatment (1.76 mg/L/hour; 95% CI= 1.47 2.06 for males and 1.73 mg/L/hour; 95% CI= 1.39 2.07 for females). From hour 12 to hour 30 of antibiotic treatment, CRP kept rising at a less rapid rate in both sexes (0.38 mg/L/hour; 95% CI= 0.17 0.58 for males and 0.37 mg/L/hour; 95% CI= 0.13 0.61 for females). On the other hand, for neonates with antibiotics started after birth, at time 0 (antibiotic start), both sexes had mean CRP values statistically different from 0 (32.3 mg/L; 95% CI= 30.36 34.24 for males and 33.33 mg/L; 95% CI 30.90 35.76 for females), but these values were considerably higher than those of the neonates who received antibiotics at birth. Unlike neonates treated at birth, those treated after birth had CRP levels that remained stable in the first 12 hours of antibiotic treatment (0.02 mg/L/hour; 95% CI= −0.16 0.20 for males and 0.27 mg/L/hour; 95% CI= 0.04 0.50 for females) and from hour 12 to hour 30 of antibiotic treatment, CRP levels started to decrease (−0.69 mg/L/hour; 95% CI= −0.82 −0.56 for males and −0.80 mg/L/hour; 95% CI= −0.96 −0.64 for females). We found no statistically significant difference between sexes in both groups (antibiotics started at birth or after), neither in intercepts nor in slopes. Figure 1 graphically represents these findings in its middle part.

Regarding neonates with antibiotics started at birth, at time 0 (antibiotic start and birth), CRPlog2 values were not statistically different from 0 (0.22 log2 mg/L; 95% CI= −0.06 0.49 for males and 0.28 log2 mg/L; 95% CI −0.04 0.60 for females). In both sexes, CRPlog2 rose in the first 12 hours of antibiotic treatment (0.32 log2 mg/L/hour; 95% CI= 0.30 0.34 for males and 0.33 log2 mg/L/hour; 95% CI= 0.30 0.35 for females). From hour 12 to hour 30 of antibiotic treatment, CRPlog2 kept rising with a less rapid rate in both sexes (0.033 log2 mg/L/hour; 95% CI= 0.026 0.041 for males and 0.027 log2 mg/L/hour; 95% CI= 0.018 0.036 for females). On the other hand, for neonates with antibiotics started after birth, at time 0 (antibiotic start) both sexes had mean CRPlog2 values statistically different from 0 (4.9; 95% CI= 4.73 5.07 for males and 4.97; 95% CI 4.75 5.18 for females) and much higher compared to the neonates with antibiotics started at birth. Unlike neonates treated at birth, in those treated after birth, CRPlog2 remained stable in the first 12 hours of antibiotic treatment (−0.004 log2 mg/L/hour; 95% CI= −0.02 0.01 for males and −0.0015/hour; 95% CI= −0.02 0.014 for females) and from hour 12 to hour 30 of antibiotic treatment, CRPlog2 started to decrease (−0.04 log2 mg/L/hour; 95% CI= −0.045 −0.035 for males and −0.04 log2 mg/L/hour; 95% CI= −0.047 −0.035 for females). In both groups (antibiotic started at birth or after), we found no statistically significant difference between sexes, neither in intercept nor in slopes. Figure 2 graphically represents these findings in its middle part.

Table 4 presents the various evolutions in CRP levels and its base 2 logarithm following CRP peak.

**Table 4:** Mixed linear regression model with random effects on intercept and slope predicting CRP and base 2 CRP logarithm in male and female neonates from CRP peak to 120 hours thereafter

Fixed effects		CRP (mg/L)				CRPlog2 (mg/L)			
		Coefficient	P-value	[95% Conf. Interval]		Coefficient	P-value	[95% Conf. Interval]	
♂	Intercept (at time of CRP peak)	32.654	<0.001	30.501	34.808	4.954	<0.001	4.868	5.039
♀		34.995	<0.001	32.352	37.638	5.041	<0.001	4.936	5.146
Delta ♀-♂		2.341	0.178	−1.069	5.750	0.087	0.208	−0.049	0.223
♂	Slope (/h)	−0.3180	<0.001	−0.341	−0.295	−0.0322	<0.001	−0.03308	−0.03138
♀		−0.3434	<0.001	−0.372	−0.315	−0.0324	<0.001	−0.03346	−0.03142
Delta ♀-♂		−0.0254	0.173	−0.062	0.011	−0.00021	0.756	−0.001536	0.001116

Random effects									
Variance of intercept		18.288		17.085	19.576	0.738		0.69	0.789
Variance of slope		0.171		0.157	0.186	0.006		0.005	0.007
Conditional intraclass correlation		0.86		0.84	0.88	0.90		0.88	0.91
Conditional R^2^		0.86				0.97			
Marginal R^2^		0.36				0.66			
Number of patients		506				506			
Number of observations		1730				1730			

Residual-fitted values diagnostic plots for both models are provided in supplementary materials section

Slopes are expressed in mg/L/hour. Intercepts are expressed in mg/L

♂: male neonates; ♀: female neonates Delta ♀-♂: difference between male and female neonates

At time 0 (CRP peak), both sexes had mean CRP values statistically different from 0 (32.65 mg/L; 95% CI= 30.5 34.81 for males and 35.0 mg/L; 95% CI 32.35 37.64 for females) with no statistically significant difference between sexes in CRP peak levels. In both sexes, CRP levels decreased (−0.32 mg/L/hour; 95% CI= −0.34 −0.29 for males and −0.34 mg/L/hour; 95% CI= −0.37 −0.31 for females) with no statistically significant difference between sexes. Figure 1 graphically represents these findings in its right part. Unfortunately, this model does not fit the data correctly, as CRP would drop below zero after 96 hours from peak. The CRPlog2 model seems much more adequate in describing the CRP dropping kinetic (residual-fitted values diagnostic plots are provided in supplementary materials). At time 0 (CRP peak), both sexes had mean CRPlog2 values statistically different from 0 (4.95 log2 mg/L; 95% CI= 4.87 5.04 for males and 5.04 log2 mg/L; 95% CI 4.94 5.15 for females) with no statistically significant difference between sexes. Therefore, we found no differences in CRP peak values between male and female neonates. In both sexes, CRPlog2 levels decreased (−0.032 log2 mg/L/hour; 95% CI= −0.033 −0.032 for males and −0.032 log2 mg/L/hour; 95% CI= −0.033 −0.031 for females) with no statistical difference between sexes. Thus, after the peak, CRP decreased by half every 31.1 hours in males and 30.9 hours in females, with no statistically significant difference. Figure 2 represents graphically these findings in its right part.

## Discussion

In our population, for every two female neonates, three males were included, reflecting a higher proportion of male neonates treated for EONS, consistent with figures reported by other authors [24]. The higher risk of jaundice in our male population can be attributed to the multiethnicity of the Brussels community, with a high prevalence of glucose-6-phosphate-dehydrogenase deficiency, a condition linked to a gene located on the X chromosome. Neonates in our population seem to present less likely preterm birth and low birth weight compared to current literature (median gestational age of 40 weeks versus 39 and birth weight of 3520 g versus 3250 g) [24]. The lower proportion of confirmed EONS compared to other authors (0% versus 375/21 703=1.7%) [24] can be explained by the characteristics of our NU, which serves as a peripheral non-referral centre, with fewer preterm and critically ill neonates. CRP and CRPlog2 values in neonates with likely but unconfirmed EONS significantly rise significantly in the first 24 hours of life, exceeding levels observed in a healthy neonatal population [5]. Interestingly, female neonates showed an earlier and more rapid increase in CRP or CRPlog2 values with higher levels at 12 hours of life, consistent with previous reports [8, 14, 15]. Both sexes seem to reach a plateau of CRPlog2 at 24 hours of life, suggesting that male and female neonates would achieve the same level of CRPlog2 at a steady state, but female neonates would reach it faster. No statistically significant sex differences were found in CRP peak, however, as this is a retrospective study, we might have failed to show the difference in CRP peak between sexes just because once CRP was observed rising earlier in female neonates, they might have experienced a faster antibiotic start.

We will now focus on the hypothised mechanism that could explain the figures we observed in our population. The first hours of an inflammatory response mainly involve innate immunity, especially in neonates, whose adaptative immunity is still immature and depends significantly on the maternal antibodies providing only passive immunity against pathogens. This innate response seems triggered by the detection of pathogen-associated molecular patterns by the Toll-like receptors (TLR) [25]. TLR activation could lead to the production of pro-inflammatory cytokines, such as the IL-6, controlling the secretion of CRP by the hepatocytes. The evidences from Chang et *al* [26] even if coming from a non-paediatric population, seem to corroborate this hypothesis. Moreover, the genes of principal protein members of the TLR signaling pathway seem to be linked to the X-chromosome [25], and polymorphisms of X-linked genes of the TLR pathway may play a role in the differences we observed. Higher CRP levels were reported in prepubertal girls with acute inflammatory processes such as pneumonia, bronchiolitis, pyelonephritis, and severe sepsis [14, 15], even though most in vitro studies showed higher IL-6 levels in men than in women after TLR stimulation [27–29]. Genes encoding some of the main proteins in the TLR signaling pathway are located on the X chromosome [25], and polymorphisms in X-linked TLR pathway genes may contribute to the differences we observed. Indeed, higher IRAK1 gene and protein expression in leukocytes has been described by others authors in female neonates, supporting that expression of TLR4 signaling kinases encoded on the X chromosome might be modified in monocytes from female umbilical cord blood [30]. That increased basal expression of IRAK1 could contribute to the higher inflammatory response and better prognosis observed in female neonates. The placental response to pathogens is another possible hypothised mechanism that could explain sex differences in the neonatal inflammatory response, as the placenta carries the identical sex chromosomes as the foetus. Analysis of the transcriptome of late first-trimester placentas from Sun et al. [31], revealed enhanced levels of genes related to immunity and cytokine-mediated signaling in female placentas.

In the first 30 hours of antibiotics, we found no sex differences in CRP and CRPlog2 over time, likely due to the effect of the treatment initiated in likely EONS. On the other hand, we found a very different pattern between neonates treated at birth and those treated after birth, especially in the first 12 hours after antibiotics start. Two non-contradictory hypotheses might explain these differences. In the first one, we assume that neonates treated at birth are more likely to be symptomatic and thus more critically ill, with a faster rise in CRP in the first 12 hours and a slower response to antibiotics failing to show a decrease in CRP levels between 12 and 30 hours after antibiotics start. This hypothesis is corroborated by the fact that neonates treated at birth had higher CRP and CRPlog2 values at birth than those treated after birth. In the second hypothesis, we assume that CRP could exhibit an exponential growth pattern until reaching a steady state. We could also assume that antibiotics do not affect CRP levels in the first 12 hours of treatment. In this scenario of assumptions, the differences observed could be explained by the fact that antibiotics have been started after reaching the steady state for neonates treated after birth and before reaching the steady state for neonates treated at birth. The fact that the slope of the CRPlog2 in the first 12 hours of life before treatment is very similar to the slope of the first 12 hours of life of neonates treated at birth corroborates this hypothesis. It suggests that the logarithmic model is more adequate in describing the rising levels of CRP over time. We found no sex differences in the CRP decreasing phase. Our findings show how CRP clearly decreases following a logarithmic pattern with a considerably longer half-life (30–31 hours) compared to the 19 hours of adults [9].

### Strengths and limitations

To our knowledge, this is the first study describing sex-specific differences in inflammation in a population with EONS. Moreover, previously described CRP values came from a primarily Caucasian healthy neonatal population [5]. Our study provides values from a multi-ethnic neonatal population with likely but unconfirmed EONS. Since prenatal infections are uncommon, we can reasonably assume that birth (time zero) marks the onset of the inflammatory process. This timing is uncertain in other studies conducted on children or neonates with late-onset infections. The monocentric, retrospective design of the study is a clear and major limitation. Another limitation is linked to the setting: this study has been conducted in a secondary centre where preterm and critically ill neonates are poorly represented. Moreover, none of the cases had a microbiological confirmation, we cannot therefore exclude the possibility that many neonates with aseptic inflammation were included in the study population. Nevertheless, we could assume that CRP’s ascending and descending pattern is likely to be the same regardless of the cause of inflammation. On the other hand, the aetiology of the inflammation certainly significantly influences the CRP evolution over time within the first 30 hours from the start of antibiotics. Accordingly, our results may primarily apply to a predominantly term neonatal population, with a small percentage of preterm neonates, in a secondary centre serving a multi-ethnic, high-income society. Moreover, the results can only be extended to neonates with likely but unconfirmed EONS (as defined in methods section).

## Conclusion

In neonates with likely but unconfirmed EONS, (as previously defined), CRP probably increases following a logarithmic pattern. Before antibiotic treatment, female neonates in our population have a statistically significant earlier rise in CRP levels. On the other hand, we found no significant differences between sexes in CRP peak levels. After reaching its peak, CRP levels decreased following a logarithmic pattern with no differences between sexes. These findings cannot yet lead to a clinical, sex-specific recommendation of different CRP timing tests and thresholds, unless prospective validation comes from some more studies with larger sample size including more preterm neonates and confirmed sepsis cases.

## Supplementary Material

Below is the link to the electronic supplementary material.

Supplementary Material 1

## Data Availability

The dataset analysed can be made available to interested researchers by the authors of this article.

## Abbreviations

CRP: C-reactive protein
CRPlog2: Base 2 logarithm of C-reactive protein
EIH: Etterbeek-Ixelles Hospital
EONS: Early onset neonatal sepsis
IL: Interleukin
LONS: Late onset neonatal sepsis
MW: Maternity ward
NS: Neonatal sepsis
NU: Neonatal unit
TLR: Toll-like receptors
