# Supplementary figures and images for: Mind the “CRP gender gap”! sex differences in CRP evolution over time in neonatal sepsis: a monocentric retrospective cohort study
Source: Biol Sex Differ. 2026 Feb 13;17:53. doi: 10.1186/s13293-026-00841-9 (PMC13005365; doi:10.1186/s13293-026-00841-9)

CRPlog2 on hours post CRP peak.

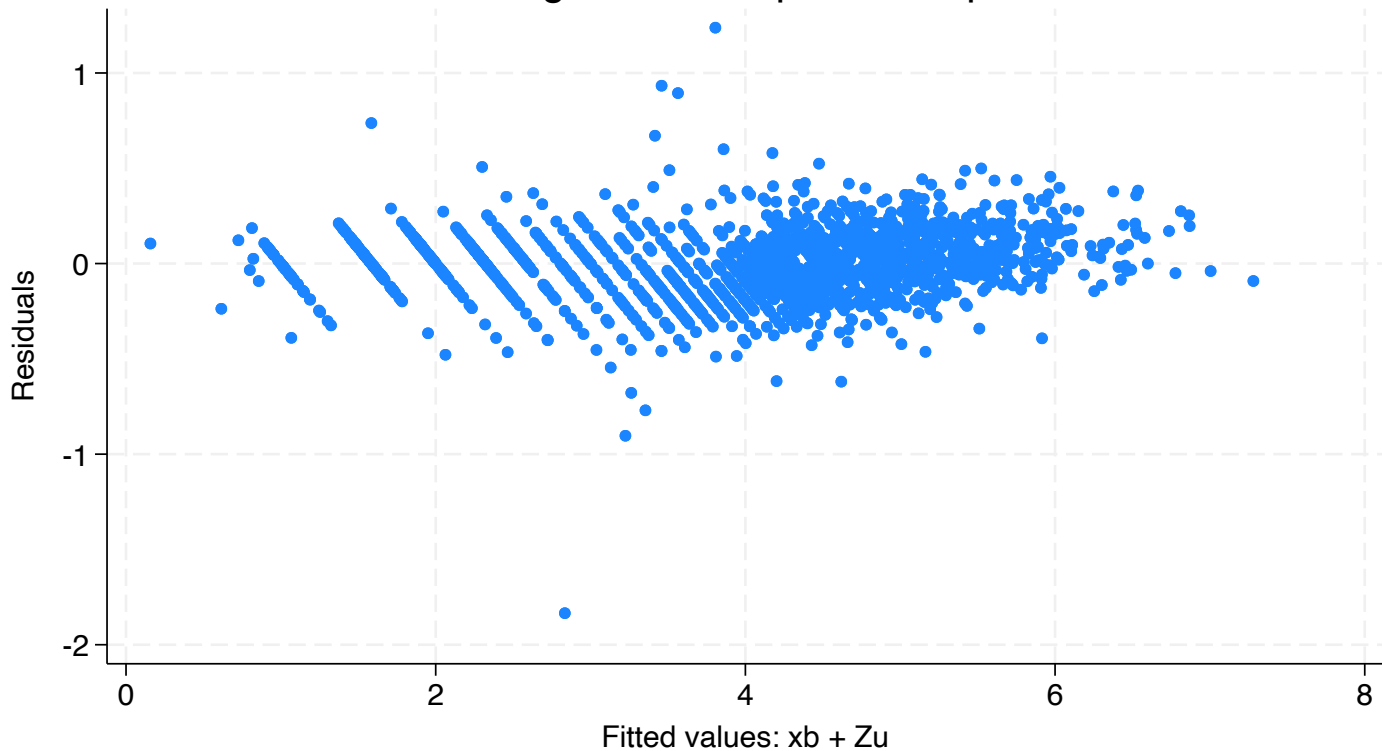

Supplement: Supplementary file 1 — Supplementary Material 1 [file 13293_2026_841_MOESM1_ESM.zip › CRPloghpostpeak.pdf]

# CRP on hours of life.

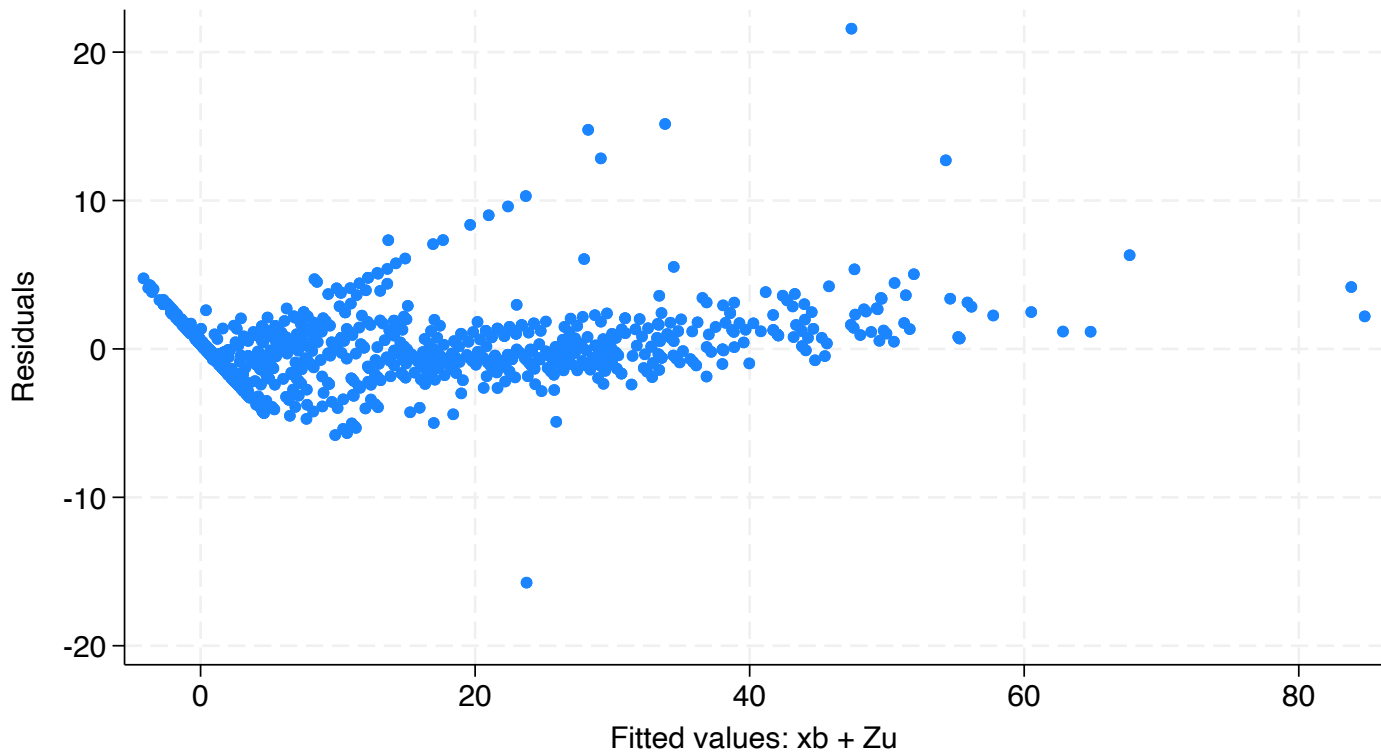

Supplement: Supplementary file 1 — Supplementary Material 1 [file 13293_2026_841_MOESM1_ESM.zip › CRPhlife.pdf]

# CRP on hours post antibiotic start.

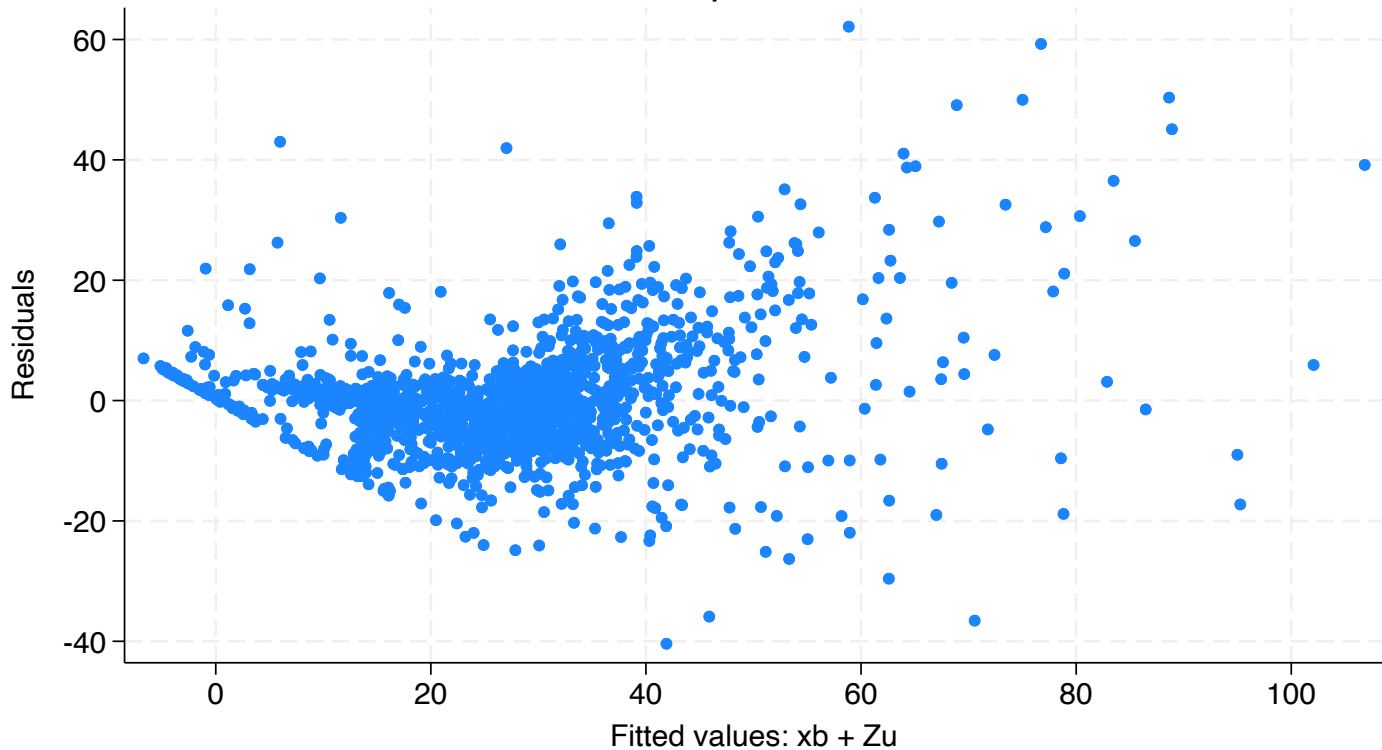

Supplement: Supplementary file 1 — Supplementary Material 1 [file 13293_2026_841_MOESM1_ESM.zip › CRPhpostab.pdf]

CRP on hours post CRP peak.

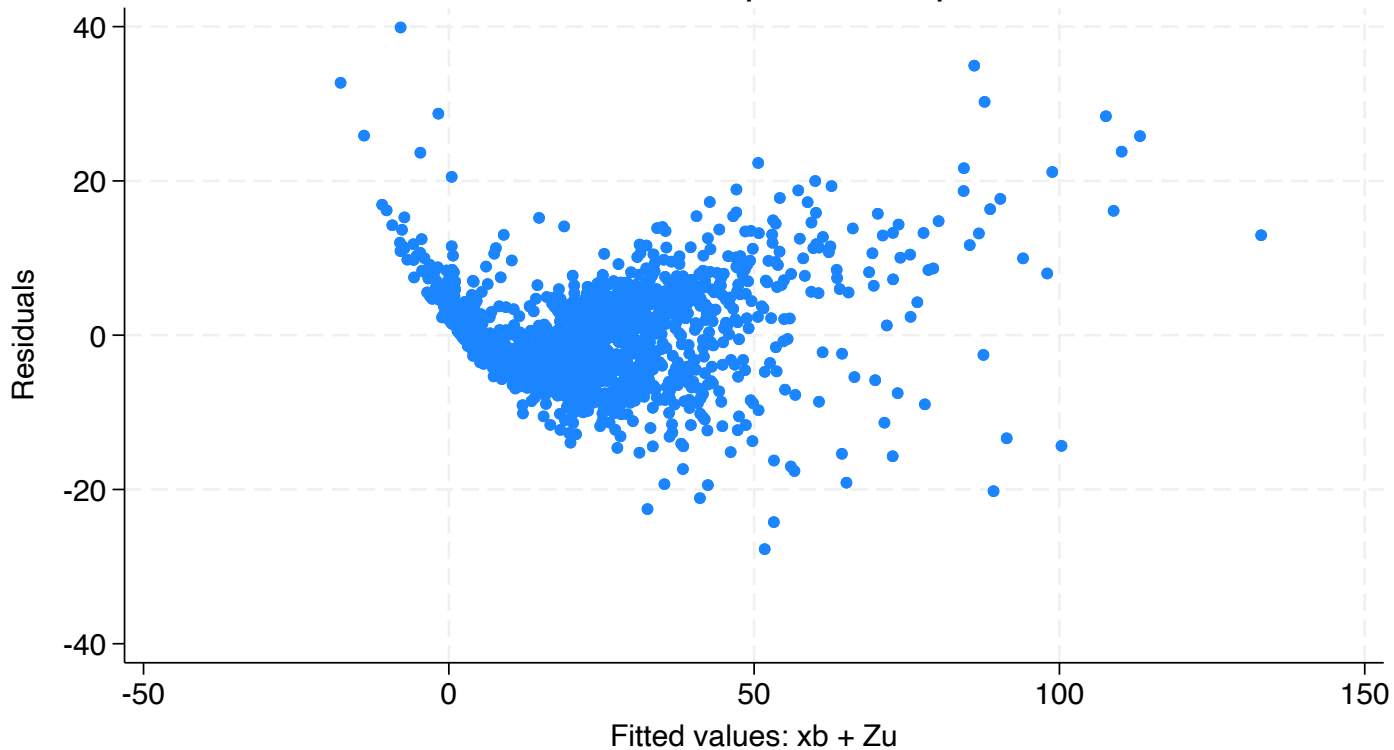

Supplement: Supplementary file 1 — Supplementary Material 1 [file 13293_2026_841_MOESM1_ESM.zip › CRPhpostpeak.pdf]

# CRPlog2 on hours of life.

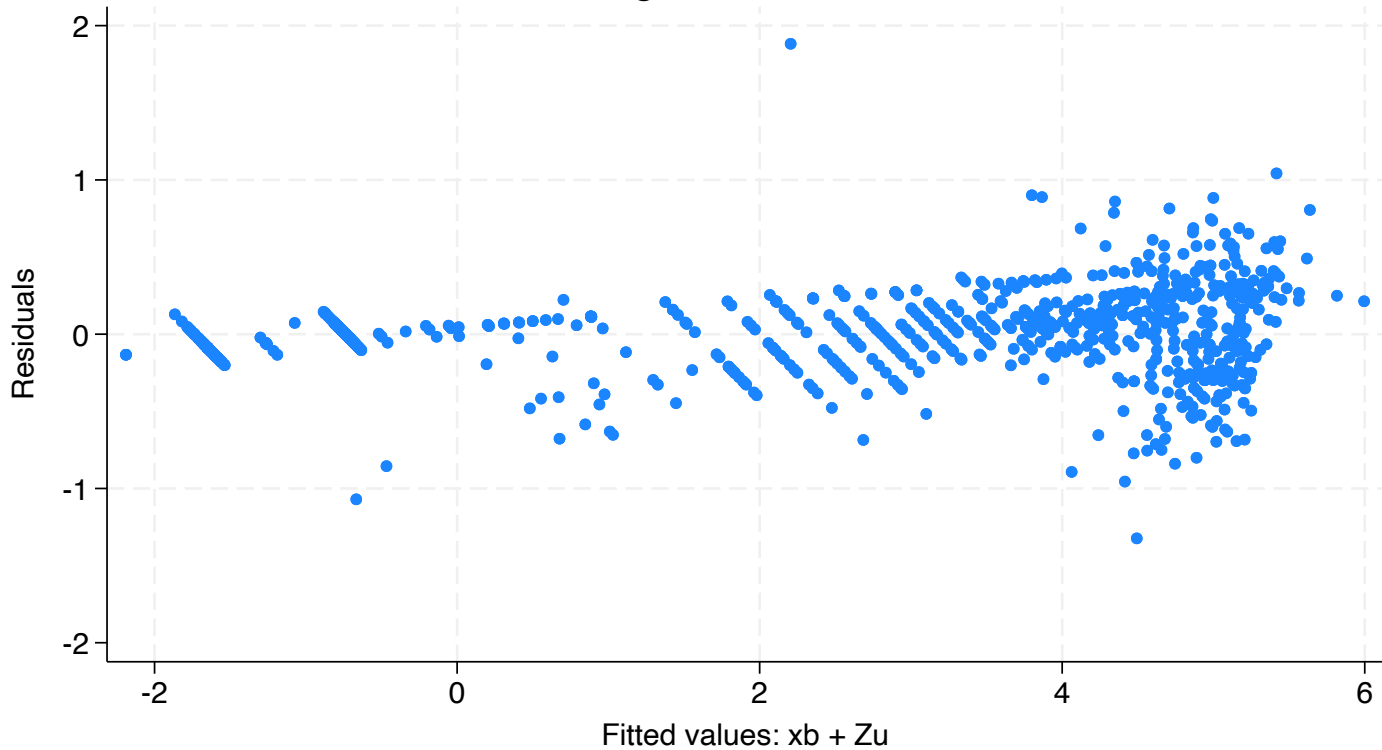

Supplement: Supplementary file 1 — Supplementary Material 1 [file 13293_2026_841_MOESM1_ESM.zip › CRPloghlife.pdf]

# CRPlog2 on hours postantibiotic start.

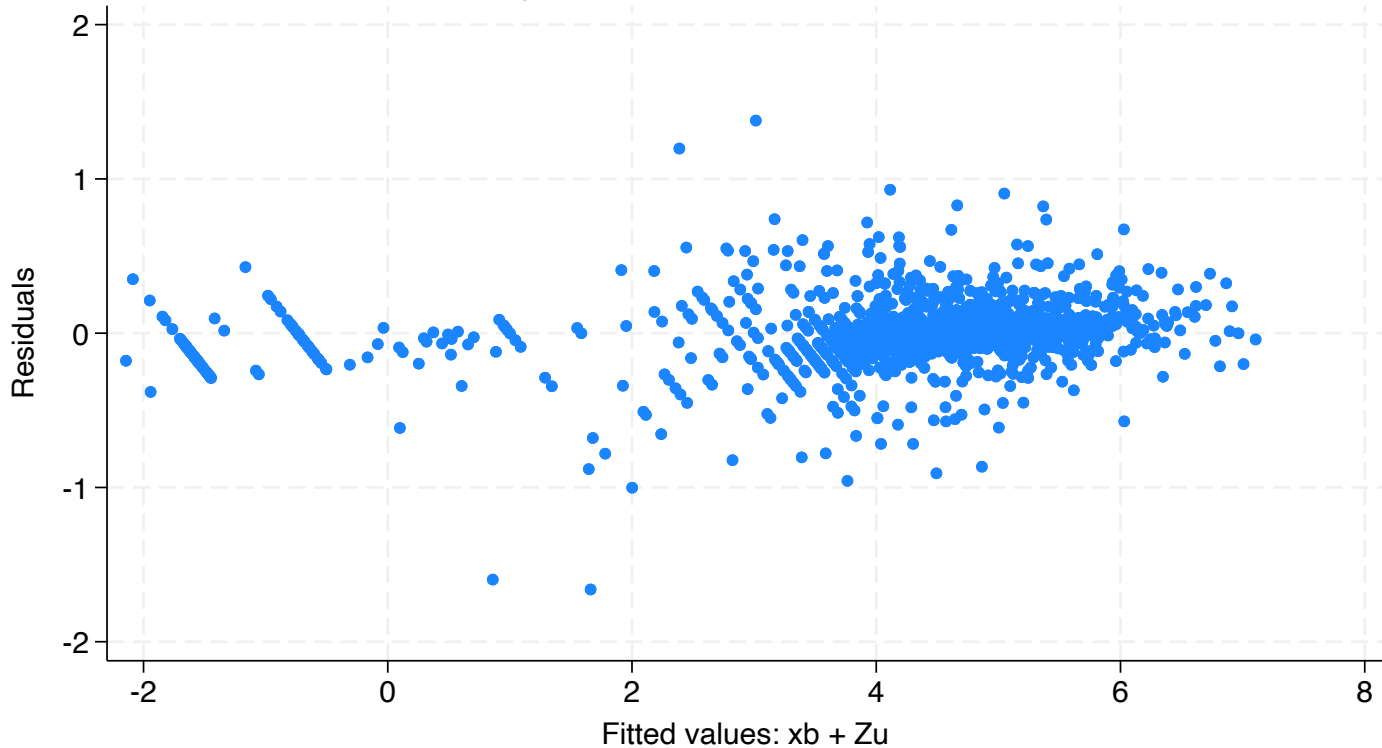

Supplement: Supplementary file 1 — Supplementary Material 1 [file 13293_2026_841_MOESM1_ESM.zip › CRPloghpostab.pdf]
